# 786. Online CME Improves Adoption of Clinical Practice related to Incorporating long-acting Injectables into HIV Clinics

**DOI:** 10.1093/ofid/ofad500.847

**Published:** 2023-11-27

**Authors:** Sara Thorpe, Donald Blatherwick, Maria B Uravich

**Affiliations:** Medscape Education, New York, New York; Medscape LLC, Medford, New Jersey; Medscape Education, New York, New York

## Abstract

**Background:**

Recently, the first long-acting injectable (LAI) for the treatment of HIV was approved by the FDA. Although this advancement in therapeutics offers many potential advantages, including eliminating the needs for daily medications, the successful uptake into clinical settings will require navigating systems-level challenges such as clinic or hospital infrastructure. This study examined whether online continuing medical education (CME) focused on evidence-based best for incorporating long-acting injectables into clinical care would result in the adoption of new clinical practices.

**Methods:**

Performance in the real world was assessed 30-60 days post-education for learners in the target audience(s). Email invitations were sent for the first 3 months of learners. Each respondent reported for each possible practice whether they were implementing for the first time or had modified it due to education, they were already doing it prior to education; or they were not doing it before or after education. They also indicated barriers they experience at least “some” of the time for each practice. The activity posted on 4/15/2022. Data was collection ended on 2/1/2023.

**Results:**

There were a total of 39 learners consisting of PCPs, OB/GYNS, Nurses and NPs. Analysis showed that 80% of learners made a practice change or had practices reinforced due to education. Top 3 practice changes included:• 59% of learners are now educating eligible patients about the availability of LAI ART• 57% are staying abreast of current data from clinical trials, evaluating LAI ART for HIV• 56% are implementing LAI ART in clinical practice

Survey results evidence persistent barriers to incorporating long-acting injectables into HIV Clinics. Top barriers included:• 38% Lack understanding of guidance about patient eligibility• 24% Lack knowledge of about best practices for getting started with LAI ART15% lack expertise in counseling patients on LAI ART

**Conclusion:**

The clinical practice changes identified in this assessment provide compelling evidence that participation in online CME/CE prompts adoption of changes in practice related to incorporating LAI ART in the care of people with HIV. Future education is needed to address the barriers identified in this assessment.

**Disclosures:**

**All Authors**: No reported disclosures

